# The role of epoxyeicosatrienoic acids in the cardiovascular system

**DOI:** 10.1111/bcp.12603

**Published:** 2015-02-05

**Authors:** L Yang, K Mäki-Petäjä, J Cheriyan, C McEniery, I B Wilkinson

**Affiliations:** Experimental Medicine and Immunotherapeutics, Department of MedicineBox 110, Addenbrooke's Hospital, Cambridge, CB2 0QQ, UK

**Keywords:** cardiovascular system, epoxyeicosatrienoic acids, soluble epoxide hydrolase inhibitor, vascular tone

## Abstract

There is increasing evidence suggesting that epoxyeicosatrienoic acids (EETs) play an important role in cardioprotective mechanisms. These include regulating vascular tone, modulating inflammatory responses, improving cardiomyocyte function and reducing ischaemic damage, resulting in attenuation of animal models of cardiovascular risk factors. This review discusses the current knowledge on the role of EETs in endothelium-dependent control of vascular tone in the healthy and in subjects with cardiovascular risk factors, and considers the pharmacological potential of targeting this pathway.

## Introduction

Cardiovascular disease remains one of the greatest challenges faced by medicine today. It is responsible for approximately 3 in 10 deaths worldwide [1]. In the UK, 1 in 6 deaths in men and 1 in 10 deaths in women are attributable to cardiovascular disease, resulting in an average of 200 deaths per day [2].

**Figure 1:**
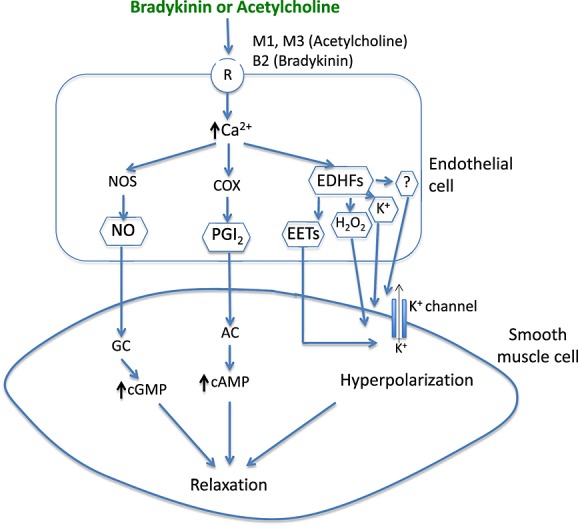
Mechanisms of endothelial dependent vasodilatation mediated by nitric oxide, prostacyclin and endothelium derived hyperpolarizing factors. Pharmacological agonists can bind to endothelial receptors and stimulate the release of these factors in a calcium dependent manner. The vasodilating factors act on the smooth muscle and mediate vasodilatation by mechanisms shown in Figure 2. R, receptor; M1 and M3, muscarinic receptors; B2, bradykinin receptor; Ca^2+^, calcium ions; NOS, nitric oxide synthase; NO, nitric oxide; GC, guanylate cyclase; cGMP, cyclic guanosine monophosphate; PGI_2_, prostacyclin; AC, adenylate cyclase; cAMP, cyclic adenylate monophosphate; EDHF, endothelium derived hyperpolarising factor; EET, epoxyeicosatrienoic acid; H_2_O_2_, hydrogen peroxide; K^+^, potassium ions

**Figure 2:**
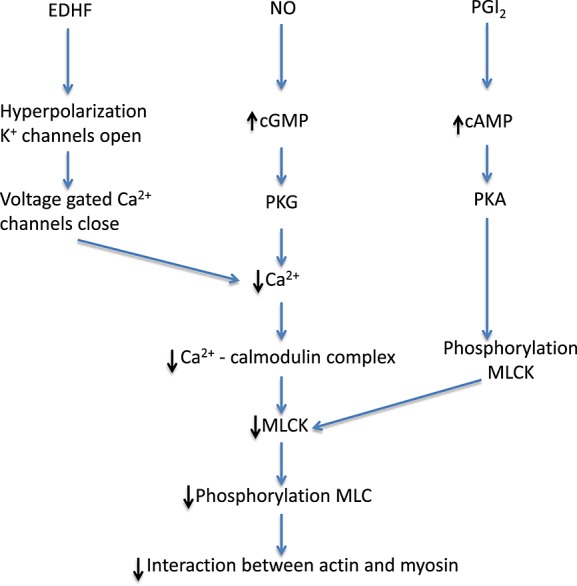
The diagram shows that both EDHF and NO mediate smooth muscle relaxation by reducing smooth muscle cell intracellular calcium, whereas PGI_2_ mediates relaxation via a calcium independent mechanism. EDHF, endothelium derived hyperpolarising factor; K^+^, potassium; NO, nitric oxide; cGMP, cyclic guanosine monophosphate; PKG, cGMP- dependent protein kinase; PGI_2_, prostacyclin; cAMP, cyclic adenylate monophospate; PKA, cAMP- dependent protein kinase; Ca_2+_, calcium; MLCK, myosin light chain kinase; MLC, myosin light chain

The vascular system is made up of approximately 60 000 miles of different-sized blood vessels, lined by a single layer of endothelial cells [3]. The pioneering work of Furchgott in the 1980s demonstrated that the endothelium not only serves as an inert lining of blood vessels, but releases endothelium-derived relaxing factors (EDRF) [4], later identified as nitric oxide (NO). It is now known that the endothelium releases many vasodilating molecules including prostacyclin (PGI_2_) [5, 6], and endothelium-derived hyperpolarizing factors (EDHF) (Figure 1) [7], and vasoconstricting molecules such as endothelin, angiotensin II and thromboxane. These regulate smooth muscle tone (Figure 2), balance anticoagulation and thrombosis, modulate immune responses, and regulate cell growth. Shear stress exerted on the vessel wall or stimulation of endothelial receptors with drugs can induce the release of endothelium-derived mediators [8]. Change in vascular tone in response to pharmacological stimulation is a reproducible ‘surrogate measure’ of overall endothelial function [9], which importantly predicts cardiovascular events in humans [10–14].

Endothelial dysfunction, characterized by an underproduction of vasodilators and an overproduction of vasoconstrictors, is a key predisposing factor to the initiation of atherosclerosis [15]. It appears early in the course of cardiovascular disease, even before the clinical manifestation of atherosclerosis or vascular disease. Traditionally, endothelial dysfunction predominantly refers to impaired NO signalling [16], but it has become evident that other endothelium-derived mediators, such as EDHF, may also be affected. EDHF (or EDHFs) describes a number of factors, including epoxyeicosatrienoic acids (EETs), hydrogen peroxide (H_2_O_2_) [17], potassium (K^+^) ions [18, 19] and likely other factors, depending on the vascular bed. It appears that larger conduit arteries have a greater expression of endothelial nitric oxide synthase (NOS), whereas the EDHF mediated pathway becomes more significant as vessel size reduces [20]. Indeed, resistance arteries with a diameter <400 µm are vital in modulating peripheral vascular resistance, and may be involved in the pathophysiology of hypertension. Increasing evidence suggests that EETs, in particular, exert cardio-protective effects in the smaller resistance vessels, and up-regulating the EETs signalling pathway pharmacologically may be beneficial in improving endothelial function. All drug and molecular target nomenclature in this review conforms to the British Journal of Pharmacology's Guide to Receptors and Channels [21].

## Synthesis and metabolism of EETs

**Figure 3:**
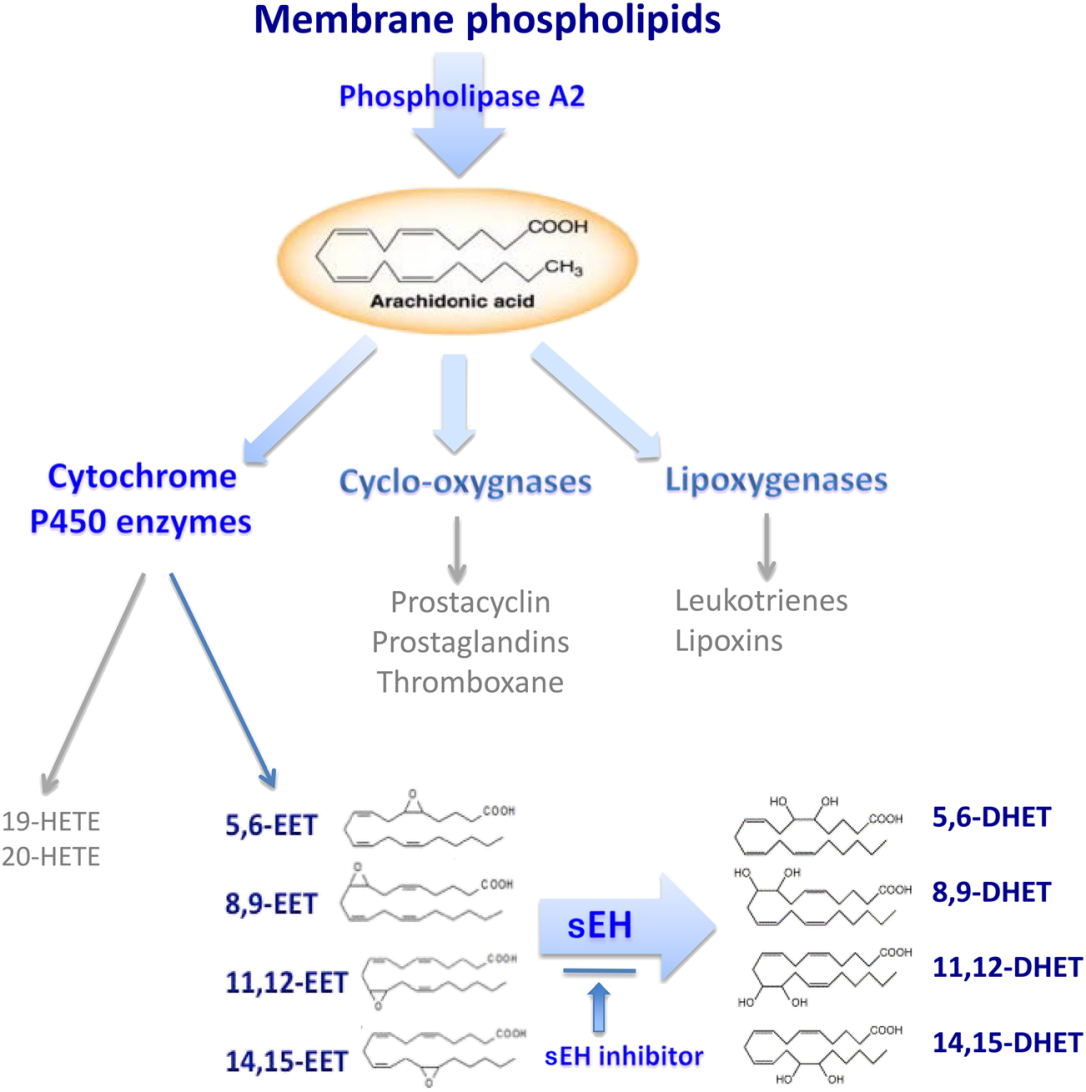
Arachidonic acid is liberated from phospholipids by phospholipase A_2_ enzyme. There are many products of arachidonic acid metabolism and EETs are products of cytochrome P450 enzymes. There are four regio-isomers of EETs. *In vivo*, the majority of EETs are readily hydrolyzed by soluble epoxide hydrolase enzymes to their corresponding DHETs. HETE, hydroxyyl-eicosatetraenoic acid; EET, epoxyeicosatrienoic acid; sEH, soluble epoxide hydrolase; DHET, dihydroxyepoxyeicosatrienoic acid

Arachidonic acid metabolism leads to the production of two vasodilating factors, PGI_2_ and EETs. EETs are the product of a number of cytochrome P450 (CYP450) enzymes. CYP2C and CYP2J produce EETs of four different isoform,: 5,6-EET, 8,9-EET, 11,12-EET and 14,15-EET (Figure 3) [22]. EETs are mainly produced by CYP2C9 and CYP2J9, although CYP2C8, CYP2C19 and CYP2J2 are also involved [23, 24]. CYP2C9 mainly produces EETs in the vascular endothelial cells, and CYP2J9 is expressed in the cardiomyocytes [25], kidneys [26], pancreas [27], lung [28] and the brain [29], though, with less catalytic activity compared with the 2C family [30]. CYP4A and CYP4F families in the vascular smooth muscle cells catalyze the ω-hydroxylation of arachidonic acid to hydroxyl-eicosatetraenoic acids (HETEs), which act as vasoconstrictors in the vascular system. Although this review mainly focuses on the role of EETs metabolized from arachidonic acid, EETs can also be generated from eicosapentaenoic acid, and mediate dilatation of microvessels with comparable potency in a similar mechanism [31].

*In vivo*, EETs are rapidly metabolized by soluble epoxide hydrolase enzymes (sEH) to their corresponding diols, dihydroxyepoxyeicosatrienoic acids (DHETs), with a short half-life of about 8 min [32, 33]. The C-terminal domain of the sEH enzyme is involved in the hydrolysis of EETs, whilst the N-terminal domain of sEH demonstrates lipid phosphatase activity. This is thought to limit the physiological effects of EETs, as they are generally more biologically active than DHETs [34], but in some vascular beds, such as canine coronary microcirculation [35] and murine mesenteric arteries [36], EETs and DHETs may be equipotent vasodilators. The substrate specificity for sEH is regio-isomer selective, e.g. 5,6-EET is a poor substrate for sEH [37, 38]. EETs are also metabolized by other pathways, including β-oxidation, ω-oxidation and chain elongation, particularly under sEH inhibition [39]. EETs can be incorporated into cell membrane phospholipids, through an acyl-coenzyme A-dependent mechanism, and liberated through the action of phospholipase A_2_ when the cell is activated [30].

## The role of EETs in regulating vascular tone

It has long been known that derivatives of arachidonic acid regulate vascular tone [40, 41]. The hypothesis that non-cyclo-oxygenase metabolites are involved in endothelium dependent regulation of vascular tone arose from experiments showing attenuated arachidonic acid induced relaxation under CYP450 inhibition [42, 43].

There is now convincing evidence that hyperpolarizing factors released from the vascular endothelium show similar characteristics to CYP450 metabolites [44] and EETs have been identified as a hyperpolarising factor in both animal [45] and human vessels [46]. The vasodilatory effects of EETs can be regio-isomer and organ selective [47]. For example, in mice mesenteric arteries, 8,9-EETs are the most potent for regulating vasorelaxation [36], whereas in rat kidneys, 8,9-EETs can be metabolized by COX enzymes to vasoconstrictor metabolites in pre-glomerular vessels [48], and in pulmonary arteries, 8,9-EETs increase pulmonary artery constriction [49].

In humans, 11,12-EETs mediate vasorelaxation in internal mammary arteries and under inhibition of NO and PGI_2_ syntheses, cytochrome P450 inhibition further reduces both bradykinin and acetylcholine stimulated flow, suggesting a role for CYP450 metabolites in agonist induced vasodilatation [46]. *In vivo*, the role of EDHFs also varies depending on the vascular bed and the mode of stimulation. In healthy subjects, there is a greater role of EDHF in bradykinin-, but not acetylcholine-induced vasodilatation. Exercise induced vasodilatation in skeletal muscles can release EETs under NOS inhibition [50], elucidating cross-talk between the various endothelial released vasodilating factors, and this may be more significant in different cardiovascular risk groups.

The physiological role of EETs in maintaining basal tone appears to be limited. Basal flow response was investigated in six human *in vivo* studies, and five reported no change in basal flow in healthy subjects [51–55], and one reported 13% and 17% reduction in response to fluconazole in 26 healthy subjects and seven patients with cardiovascular risk factors, respectively [56]. Three of these *in vivo* studies assessed basal tone in fewer than 10 subjects [51–53], and thus were significantly underpowered.

## Mechanisms of action in the vascular system

A number of different pathways are involved in mediating EET-induced vasodilatation, including calcium-dependent K^+^ channels, gap junctions, endothelial NOS and transient receptor potential (TRP) channels. The precise pathway(s) involved depends on the vascular bed, and can be endothelium dependent via intermediate-conductance calcium-dependent K^+^ (IK) and small-conductance (SK) channels, TRP channels [8, 57] leading to NOS activation [36], or through a smooth muscle effect via TRP channels or a G-protein coupled receptor, and acting via large conductance (BK) channels.

Calcium-dependent K^+^ channels on endothelial and smooth muscle cells are usually activated in a calcium-dependent fashion. K^+^ influx and hyperpolarization of the cell membrane leads to calcium channel closure on smooth muscle cells and vasorelaxation occurs as a result of reduction in intracellular calcium (Figure 2) [58].

In porcine [59] and bovine coronary arteries [60], EETs can act locally on the endothelial IK and SK channels. This interaction with calcium-dependent K^+^ channels may be through TRP channels.

TRP channels, particularly TRPV4 in the vallinoid subfamily, interact with EETs and regulate vascular tone [61, 62]. TRPV4 is a calcium permeable voltage gated channel expressed in a range of tissues including the endothelial and the smooth muscle cells. In mice, inhibition of TRPV4 with ruthenium red significantly reduces vasodilatation in CYP2C9 over-expressed arteries. Co-inhibition of EET synthesis and TRPV4 does not have an additive inhibitory effect, suggesting that EETs act primarily through the TRPV4 pathway [63]. Under NO and PGI_2_ inhibition, 11,12-EETs elicit hyperpolarization in mesenteric arteries in wild type mice, but not TRPV4−/− mice, and this can be completely inhibited by blocking IK, SK and BK channels with charybdotoxin, apamin and iberiotoxin, respectively [64]. Blood pressure is higher in TRPV4 −/− mice, suggesting that TRPV4 may be an important regulator of vascular tone.

**Figure 4:**
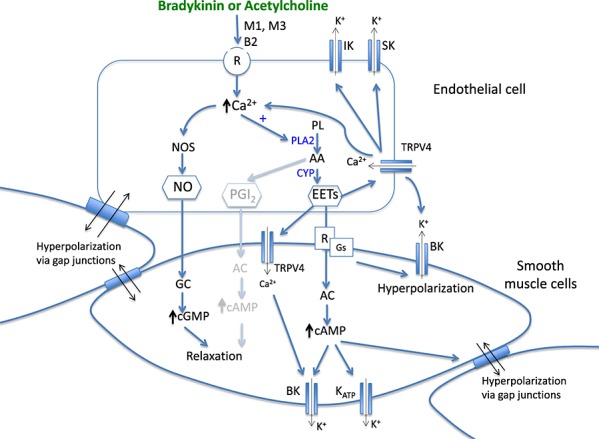
This diagram shows the mechanisms by which EETs exert hyperpolarization effects on the endothelial cell and the smooth muscle cell. Agonist binding to a luminal receptor of the endothelial cell activates phospholipase A in a calcium dependent manner, which converts phospholipids to arachidonic acid. EETs are products of CYP450 enzyme metabolism. EETs may activate the IK_Ca_ and SK_Ca_ channels via TRPV4 channels. EETs may activate BK_Ca_ and K_ATP_ channels via an EET receptor or via TRPV4 channels. R, receptor; M1 and M3, muscarinic receptors; B2, bradykinin recetor; Ca^2+^, calcium ions; NOS, nitric oxide synthase; NO, nitric oxide; GC, guanylate cyclase; cGMP, cyclic guanosine monophosphate; PL, phospholipids; PLA2, phospholipase A2; AA, arachidonic acid; CYP, cytochrome P450 enzymes; K^+^, potassium ions; BK, large conductance calcium-dependent potassium channel; K_ATP_, ATP sensitive potassium channel; TRP, transient receptor potential channels, R_GS_, G-protein coupled receptor coupled; cAMP, cyclic adenylate monophosphate

TRPV4 agonists and 11,12-EET can activate TRPV4 channels in a cluster fashion and leverage a large calcium influx through each TRPV4 channel, leading to activation of IK and SK channels [8]. The current is then likely to spread through myoendothelial gap junctions resulting in relaxation [65–68]. When vessels are stimulated with bradykinin, other TRP channels are activated, transient receptor potential cation (TRPC) channel 3 and 6. Bradykinin-induced calcium influx can be inhibited by CYP inhibitors and EET antagonists, and enhanced by a sEH inhibitor [69]. TRP channels rapidly translocate to caveolae to modulate calcium influx in response to 11,12-EETs [69]. This process is dependent on the activation of cAMP-dependent protein kinase and may be dependent on caveolin-1 [70]. In some vascular beds, an increase in intracellular calcium stimulates endothelial NOS (Figure 4) [36, 71].

In human internal mammary arteries [46], EETs act on the BK channels expressed on smooth muscle cells, and this may be via TRPV4 channels or through a specific EET receptor. TRPV4 channels mediate the activation of BK by forming a signalling complex with ryanodine receptors and BK channels on the smooth muscle.

It appears that EET activation of BK channels is not simply by binding to an extracellular domain, but there are strict requirements for their vascular activity. In bovine coronary arteries, 14(S),15(R)-EET, but not 14(R),15(S)-EET increases BK channel activity [72], whereas 11(R),12(S)-EET is the isomer that activates the BK channel in rat renal smooth muscle cells [73]. Furthermore, tethering 14,15-EET to silica beads restricts entry into smooth muscle cells, but does not attenuate its inhibitory effect on aromatase [74]. This suggests that there is a specific EET binding site on the smooth muscle cell (Figure 4). A high affinity binding site has been characterized using radioligands in U937 monocyte membranes, where a novel radiolabelled EET agonist bound in a specific, saturable and reversible manner, resulting in the production of cAMP production with similar potency as 11,12-EET and 14,15-EET. The G-protein analogue GTPγS inhibited this binding, suggesting that EETs act via a G-protein coupled receptor [75, 76]. However, a group of 47 known receptors were screened for the ability of EET regio-isomers to displace high affinity radioligands, and none was identified as a receptor for EETs [77].

In bovine [78] and porcine [79] coronary smooth muscle cells, EET-mediated smooth muscle BK activation requires intracellular GTP, but not ATP, and can be blocked by a G protein inhibitor or antibodies against Gα_s_, suggesting that a G protein is required for EETs to activate BK channels. EETs promote GTP binding to Gα_s_ in endothelial cells [80], and BK channels can be activated directly by GTP-activated Gα_s_ through a membrane-delimited action of Gα_s_, or by activation of a classic signalling cascade. In both bovine endothelial cells and U937 monocytes, EETs activate adenylyl cyclase and protein kinase A [75, 80–82], which can stimulate transmission of hyperpolarization through gap junctions [83]. In a similar fashion to activation of BK channels, EETs can activate ATP-sensitive K^+^ channels on smooth muscle cells in rats [84, 85].

## Other physiological roles of EETs

Other than mediating vascular tone, EETs modulate calcium channels on cardiomyocytes [86, 87] and 11,12-EETs can improve recovery of cardiac contractile function following prolonged ischaemia [88]. EETs also regulate pancreatic β-cell function, where 5,6-EETs directly induce insulin secretion [89], and CYP2J is highly expressed in the cells of islets to produce a significant amount of EETs in human and rat pancreas [90].

EETs attenuate inflammatory processes, which play a key role in the pathophysiology of cardiovascular diseases [91]. Various stimuli, such as microorganisms, lipid products or hypoxia can cause vascular injury and lead to endothelial activation, a highly dynamic and complex process that intertwines endothelial dysfunction and inflammation. Leukocyte-endothelial adhesion and subsequent leukocyte transmigration across the endothelium are primary events in the vascular inflammatory process influencing the initiation of atherosclerosis and cardiovascular diseases. 11,12-EET can attenuate endothelial activation and leukocyte adhesion in induced models of inflammation by inhibiting nuclear factor-kappaB (NFκB), a central mediator of this process [92].

Increased recognition of the benefits of EETs has revealed a worrying paradox that is their broad physiological impact may potentially have deleterious effects too. EETs promote endothelial cell survival by pro-angiogenetic [93] and anti-apoptotic mechanisms [94]. They contribute to vascular endothelial growth factor (VEGF) mediated stimulation of angiogenesis [95]. Whilst this may exert some protective benefits in preserving endothelial function and promoting neovascularization in ischaemic tissues [96], their potential to promote cancer metastases warrants careful consideration [97, 98]. Indeed, inhibition of CYP-derived EET synthesis increases tumour cell apoptosis, and decreases tumour growth and metastases [99].

## EET signalling in cardiovascular disease

Dysregulated EET signalling pathways may be implicated in a number of disease states. Whilst most cardiovascular risk factors are associated with impaired EETs and induction of sEH expression, there is much crosstalk between the endothelial factors, and alteration in EET signalling may change as cardiovascular disease progresses. In the presence of stable coronary atherosclerotic disease, where there is reduced NO signalling [100], EETs may in fact be up-regulated to compensate for the overall endothelial dysfunction. The first study to quantify plasma concentrations of EETs in patients with stable coronary atherosclerosis reported that subjects with ≥50% stenosis in at least one major epicardial coronary artery had significantly higher total EETs compared with healthy controls. However, within the group of patients with coronary artery disease, obese subjects had lower plasma concentrations of total EETs [101]. This is consistent with preclinical models of obesity [102, 103], suggesting a decreased CYP450 and increased sEH expression, and the overall increased EETs in subjects with coronary artery disease may be a compensatory response to the presence of advanced cardiovascular disease. Furthermore, within the group of subjects with stable coronary atherosclerotic disease, those with comparatively higher sEH activity exhibit higher levels of inflammatory molecules, such as cellular adhesion molecules, and therefore may be predisposed to more advanced vascular inflammation [104]. Thus, sEH inhibition in these higher risk subjects may represent an effective secondary prevention strategy. Although no association between flow mediated dilatation (FMD) and plasma concentrations of EETs has been observed in subjects with coronary artery disease, bradykinin-induced changes in forearm blood flow may be more reflective of EET associated microvascular function [56, 105] and more predictive of cardiovascular outcome [10]. Interestingly, the cytochrome P450 inhibitor sulfaphenazole enhances acetylcholine-induced flow in patients with coronary artery disease and this may be due to a reduction in the generation of reactive oxygen species by CYP2C in endothelial cells, thus improving NO bioavailability [53, 106].

Diabetes and obesity are associated with reduced expression of CYP2C enzymes in mice and rat models [107–109], and increased expression of CYP4A and sEH [110, 111]. Inhibition and genetic deletion of sEH can augment pancreatic EET concentrations, and prevent hyperglycaemia in diabetic mice [112]. EDHF activity appears to be impaired in different animal models of type 1 [113] and type 2 diabetes [114, 115]. In insulin resistant rats, chronic feeding of miconazole (CYP inhibitor) had no effect on mesenteric artery relaxation, whereas phenobarbital (CYP inducer) restored EDHF mediated relaxation [116]. Type 1 diabetic mice up-regulate the sEH mRNA and have lower concentrations of EETs in the brain, associated with worse stroke outcome [117]. Interestingly, one study reported increased EDHF mediated vasodilatation in femoral and mesenteric arteries of type 1 diabetic rats [118] and this was thought to be a compensatory mechanism for impaired NO production [119]. There are no human studies as yet which assess EET mediated endothelial function in diabetic patients.

In essential hypertension, there is certainly some alteration in EET signalling but its role in modulating human vascular function remains somewhat unclear. In animal models, an infusion of angiotensin II elevates blood pressure, and stimulates 20-HETE synthesis in renal microvessels [120], and decreases EETs by down regulating CYP450 epoxygenases, and increasing sEH activity [121]. In spontaneously hypertensive rats, sEH expression is elevated [122]. In humans, plasma concentrations of EETs are reduced in women with pregnancy-induced hypertension [123] and in subjects with renovascular hypertension [124]. This may be a result of reduced EET synthesis by CYP450 enzymes, and increased EET metabolism by sEH enzymes [124]. Another group reported no difference in basal plasma concentrations of EETs between healthy control subjects and hypertensive patients, but an impairment in induced EET release, in combination with NO and reactive oxygen species balance, and the endothelin-1 pathway contributed to endothelial dysfunction of conduit arteries (measured by flow mediated dilatation) in essential hypertensives [125]. The same group later demonstrated that inhibition of CYP450 reduced basal conduit arterial diameter only in healthy subjects, and not in essential hypertensives, but it did not change resistance arterial flow in both groups [55]. This is consistent with another study, which reported that a CYP450 inhibitor did not change basal flow within both normotensive and hypertensive patients, but conversely, it significantly blunted both acetylcholine and bradykinin induced flow only in hypertensives. The authors attributed this to EETs compensating for impaired NO activity in the hypertensive group [54]. Thus, it remains unclear whether there is true functionally important EET impairment in hypertensives, and in order to elucidate this, larger studies combining quantitative measure of plasma EETs and vascular function assessment would be required.

Smoking has a synergistic effect with sEH polymorphisms coding for enhanced sEH activity and thus reduced EET signalling [126] and may initiate pulmonary vascular impairment through direct injury of endothelial cells or release of inflammatory mediators [127]. Chronic injury leads to some of the vascular impairment observed in chronic obstructive pulmonary disease (COPD), such as reduced NOS and EDHF *in vitro* in pulmonary vessels [128], worsening with the progression of disease [129]. A quantitative study showed 8,9-EETs are significantly reduced in the breath condensate of COPD patients [130], and one study showed no difference in bradykinin induced vasodilatation in resistance vessels between COPD patients and other healthy smokers, though not assessing the role of EETs directly [131]. This may be an interesting group to explore and target therapeutically for the vascular and anti-inflammatory effects of EETs, as a subset of COPD patients is of a systemic inflammatory phenotype [132] associated with a three-fold elevated risk of cardiovascular admissions [133]. It is estimated that approximately 30% of COPD patients die from cardiovascular disease.

In hypercholesterolaemia, EETs may be up-regulated to compensate for an impaired NO pathway. In cholesterol fed animals, EDHF is maintained, while NO is reduced [134] and only cholesterol-fed rabbits synthesize EETs in the aorta [135]. *In vivo*, there appears to be enhanced EDHF activity in hypercholesterolaemia where there is NO deficiency [56]. It is possible to speculate that some of the EDHF activity may be secondary to EET signalling, thus suggesting that the mechanism by which EETs act, i.e. through hyperpolarization, or via the NO signalling pathway, may be dependent on the health condition of the subject.

A summary of the *in vivo* studies investigating endothelial function and the EET pathway in the healthy and diseased subjects are shown in Table 1. Current evidence suggests that EET signalling may be differentially impaired in patients with cardiovascular risk factors associated with endothelial dysfunction. EETs may become up-regulated in patients with advanced coronary artery disease, suggesting that there may be a role for targeting EET impairment early to prevent disease progression.

**Table 1:** Human *in vivo* studies using venous occlusion plethysmography with an intra-arterial infusion of a cytochrome P450 inhibitor (inhibit EET synthesis) to investigate EET-mediated regulation of vascular tone in basal flow and agonist induced vasodilatation

Author	Subjects (n)	Agonists	Inhibitors	Main findings
Halcox *et al*. [51]	Healthy subjects (*n* = 47)	Bradykinin 100, 200, 400 ng min^–1^	Miconazole 0.0125, 0.0375, 0.125 mg min^–1^	Miconazole did not change basal flow
		Acetylcholine 15, 30 µg min^–1^	Aspirin 1 g intravenous	Miconazole did not reduce acetylcholine induced flow.
		SNP 1.6, 3.2 µg min^–1^	LNMMA 4 µmol min^–1^	
Passauer *et al*. [52]	Healthy male subjects (*n* = 11)	Bradykinin 20, 40, 80 pmol min^–1^	Ibuprofen 1200 mg oral	Sulphaphenazole did not change basal flow.
			Sulphaphenazole 0.02, 0.2, 2, 6 mg min^–1^	No inhibitory effect of sulphaphenazole on bradykinin induced flow under NO inhibition.
			LNMMA 4 µmol min^–1^	
Taddei *et al*. [54]	Healthy subjects (*n* = 36)	Acetylcholine 0.15–15 µg min^–1^	LNMMA 100 µg min^–1^	Sulfaphenazole did not change basal flow.
	Essential hypertensives (*n* = 32)	Bradykinin 5–50 ng min^–1^	Sulfaphenazole 0.3 µg min^–1^	In normotensives, sulfaphenazole was did not inhibit acetylcholine or bradykinin induced flow.
	.	SNP 1–4 ng min^–1^		In hypertensives, sulfaphenazole inhibited bradykinin induced vasodilatation more than that of acetylcholine.
Bellien *et al*. [55]	Normotensive controls (*n* = 14)	None	Fluconazole 0.4 µmol min^–1^	Fluconazole had no effect on basal flow in both groups.
	Untreated essential hypertensive patients (*n* = 14)		LNMMA 8 µmol min^–1^	In normotensives, radial artery diameter reduced by fluconazole, LNMMA, and their combination.
				In hypertensives, radial artery diameter was not reduced by fluconazole.
Fichtlscherer *et al*. [53]	Healthy subjects (*n* = 5)	Acetylcholine 20, 40 µg min^–1^	Sulfaphenazole 0.2, 2 mg min^–1^	Sulfaphenazole had no effect on basal flow.
	Patients with angiogram diagnosed coronary artery disease (*n* = 16)	SNP 4, 8 µg min^–1^	LNMMA 8 µmol min^–1^	Sulfaphenazole significantly enhanced acetylcholine induced flow in patients.
Ozkor *et al*. [56]	Healthy subjects (*n* = 103)	Bradykinin 100, 200, 400 ng min^–1^	Fluconazole 0.4 µmol min^–1^	Fluconazole reduced basal blood flow, and addition of TEA further reduced blood flow.
	Normotensive with multiple cardiovascular risk factors (*n* = 71)	Acetylcholine 7.5, 15, 30 µg min^–1^	LNMMA 8 µmol min^–1^	In healthy group, TEA inhibited bradykinin induced vasodilatation but not acetylcholine.
		SNP 1.6, 3.2 µg min^–1^	TEA 1 mg min^–1^	In hypercholesterolaemics, TEA inhibited bradykinin and acetylcholine induced flow.
			Aspirin 975 mg oral	
Lee *et al*. [150]	Healthy subjects with *EPHX2* Lys55Arg and Arg287Gln polymorphisms	Bradykinin 100, 200, 400 ng min^–1^	None	Reduced bradykinin induced vasodilatation in subjects with Lys55Arg (high sEH activity) in White Americans.
	White American (*n* = 198)	Methacholine 3.2, 6.4, 12.8 µg min^–1^		
	Black American (*n* = 67)	SNP 1.6, 3.2, 6.4 µg min^–1^		

## Genetic polymorphisms

Polymorphisms exist for both the CYP450 families involved in EET synthesis and sEH enzymes required for EET metabolism. CYP2J2 gene cloning and sequence analysis revealed a range of polymorphisms, with the commonest being the G-50 T single nucleotide polymorphism (SNP). The G-50 T SNP is in the proximal promoter of CYP2J2 gene, which regulates basal transcriptional activity. The polymorphism is found in approximately 17% Africans, 13% Asians and 10% of Caucasians and is associated with lower EET activity and an increased risk of coronary artery disease [136]. Furthermore, CYP2J2 polymorphism may be an independent risk factor for the premature onset (<45 years old) of myocardial infarction (MI) in the Chinese Han population [137], and it has a synergistic effect with smoking, increasing the risk of MI by approximately 6.7 fold compared with non-smoker wild types. In type 2 diabetes, the frequency of the CYP2J2 G-50 T polymorphism is significantly higher in younger onset diabetics (<40 years) and is associated with lower plasma EET concentration [138]. A variant of CYP4A11, which oxidizes arachidonic acid to 20-HETE is associated with hypertension [139]. CYP2C9 [140] and CYP2C19 [141] polymorphisms may be associated with hypertension in the Chinese population. Interestingly, CYP2C19 plays a key role in activating clopidogrel, and polymorphisms may determine the prognosis in young patients who are receiving clopidogrel treatment following MI [142].

Multiple reports have demonstrated an association between sEH gene polymorphisms and coronary artery disease [143, 144] and cerebrovascular disease [145–147]. The human sEH gene, *EPHX2*, is localized to chromosome 8p21-p12, enclosing 19 exons. A number of polymorphisms have been identified, including variants with higher (Lys55Arg) and lower (Arg287Gln) sEH activities *in vitro* [148]. In African American subjects selected from the Coronary Artery Risk Development in young Adults (CARDIA) study, although coding for lower sEH activity *in vitro*, a positive association was found between Arg287Gln and subclinical atherosclerosis defined by coronary artery plaque calcification, with no influence on blood pressure [143]. This was attributed to EETs increasing intracellular calcium concentration in vascular smooth muscle cells [149]. Another study genotyped 2065 subjects (1085 with incident coronary heart disease and 980 non-cases) selected from the Atherosclerosis Risk in Communities (ARIC) study, and reported Lys55Arg was associated with higher sEH activity *in vivo*, and greater risk of incident coronary heart disease in Caucasians [144]. Lys55Arg genotype is also associated with reduced vasodilator response to bradykinin in Caucasian Americans [150].

## Pharmacological target

The cardioprotective benefits of up-regulating EET signalling have been elucidated by genetic and pharmacological modulation of this pathway. Deficiency in the sEH gene reduced EET metabolism and improved endothelial function [151], glucose homeostasis [152] and protected against experimental models of cerebral ischaemia [153]. Successful EET analogues act on a similar signalling pathway as endogenous EETs via the K^+^ channel, and cause vasodilator effects in bovine coronary arteries [154, 155]. In particular, one 11,12-EET analogue has the potential to reduce blood pressure *in vivo* in spontaneously hypertensive rats [156], but this has not progressed into humans yet. EET analogues may also exhibit some anti-inflammatory benefits in addition to antihypertensive effects [157, 158].

Novel sEH inhibitors developed with the aim of reducing EET metabolism have been the most progressive pharmacological agent. Older generations have weak inhibitory effectiveness and poor stability, but the newer agents are competitive, tight-binding inhibitors with nanomolar *K*_i_ values, which interact stoichiomerically with purified recombinant sEH [159]. In animal models of atherosclerosis, sEH inhibition can reduce atherosclerotic plaque lesions by up to approximately 50% in mice aortae [160, 161]. In rats with induced myocardial ischaemia and hypertension, it has the potential to reduce blood pressure [162, 163] and infarct size independent of NO [162]. In mice with induced renovascular hypertension, sEH inhibition restores the functional role of EETs in endothelium-dependent relaxation, allowing an attenuation of blood pressure, cardiac hypertrophy and prevention of coronary endothelial dysfunction [164]. Interestingly, in rats with induced malignant hypertension, the antihypertensive and renoprotective effects of sEH inhibition can be completely abolished by NO inhibition, suggesting the benefits of sEH inhibition in this condition may be dependent on the endogenous bioavailability of EETs and NO [165].

Other observed benefits of sEH inhibition include amelioration of the metabolic syndrome [166], anti-inflammatory properties [167] and protection against ischaemic stroke [168, 169]. One sEH inhibitor (AR9281) improved endothelial function in mice models of diabetes, hypertension and obesity, and significantly reduced fasting plasma glucose [170]. Whilst the same compound was well tolerated in healthy subjects in a phase 1 trial, it was terminated at phase 2 due to lack of efficacy in patients with hypertension and impaired glucose tolerance (http://clinicaltrials.gov/show/NCT00847899) [171].

In rats, sEH inhibition can improve lung function, and attenuate smoking related inflammation and emphysematous changes [172]. One concern is that in the EET pathway can enhance acute hypoxic pulmonary artery vasoconstriction in mice isolated lungs, and thus possibly contribute to the development of pulmonary hypertension, but chronic treatment with sEH inhibition for 4 months did not affect muscularization of the pulmonary vasculature and exercise tolerance. It is thought that the C-terminal epoxide hydrolase of the sEH enzyme plays a lesser role in the regulation of pulmonary resistance and morphology compared with the N-terminal phosphatase [173]. Repeat dose oral administration of a potent sEH inhibitor (GSK2256294A) attenuated lung inflammation in mice exposed to cigarette smoke [174]. The authors of this review have been involved in the phase 1 clinical trial of GSK2256294 to assess its safety, tolerability and pharmacokinetics of single and repeat doses in healthy and obese smokers (http://clinicaltrials.gov/ct2/show/NCT01762774). The pharmacodynamic effects of this drug will be assessed by venous plethysmography at baseline, after acute dosing (day 1) and after chronic dosing (day 14).

Dual action compounds which act as an EET analogue and sEH inhibitor are also under development. The extent of enzyme inhibition is dependent on the structure, and vascular relaxation has been demonstrated in bovine coronary arteries [175]. In mice with the metabolic syndrome phenotype, an EET agonist/sEH inhibitor increased vascular EET concentrations, lowered blood pressure, prevented weight gain, increased insulin sensitivity and restored acetylcholine stimulated vessel relaxation [176]. Interestingly, dual inhibition of cyclo-oxygenase 2 and soluble epoxide hydrolase may have synergistic anti-angiogenic and anti-cancer activity [177] Thus, progression of dual action agents may be a more enlightening route to unravel and balance the controversy between up-regulating EETs and their effects on cancer activity.

## Conclusion

In the last couple of decades, the broad biological effects of EETs have gained greater recognition. The beneficial role of EETs in maintaining vascular tone, modulating inflammatory responses and mediating endothelial cell growth has propelled the development of basic and clinical pharmacological research focusing on this pathway, though this is not without some challenges considering the current lack of an identified membrane protein target for EETs. Nevertheless, the need for novel compounds to impact on the pathophysiology of cardiovascular disease remains and current research is focused on up-regulating EETs with sEH inhibitors. As impairment in EET signalling is not universal across all cardiovascular risk factors, it would be worth stratifying a group of people with the most impaired EETs to target. Theoretically, augmenting EETs with an sEH inhibitor in an ideal population should enhance their cardioprotective effects, and this may be an exciting and promising route to impact on endothelial dysfunction, a disease process thought to appear early in the development of atherosclerosis, but this is not without potential risks, and certainly warrants large scale clinical trials to demonstrate efficacy.

## Competing Interests

All authors have completed the Unified Competing Interest form at http:http://www.icmje.org/coi_disclosure.pdf (available on request from the corresponding author) and declare LY had grants and fees from the Wellcome Trust Translational Medicine and Therapeutics programme in collaboration with GlaxoSmithKline, JC and IBW had support and grants from GlaxoSmithKline and KM and CM report no support from any organiszation for the submitted work. There are no financial relationships with any organizations that might have an interest in the submitted work in the previous 3 years and no other relationships or activities that could appear to have influenced the submitted work.

Dr Lucy Yang is funded by the Wellcome Trust TMAT programme, the Sackler fellowship, and Clare College Research Expenses Fund. Professor Ian Wilkinson and Dr Joseph Cheriyan are both funded by the Cambridge Biomedical Research Centre. Professor Ian Wilkinson and Dr Carmel McEniery are both funded by the British Heart Foundation.

We would like to thank Ms. Naomi Morris for her help in improving the image quality of the figures.
